# Dietary Polyphenols and Gut Microbiota Cross-Talk: Molecular and Therapeutic Perspectives for Cardiometabolic Disease: A Narrative Review

**DOI:** 10.3390/ijms25169118

**Published:** 2024-08-22

**Authors:** Raquel Cano, Valmore Bermúdez, Nestor Galban, Bermary Garrido, Raquel Santeliz, Maria Paula Gotera, Pablo Duran, Arturo Boscan, Ana-Karina Carbonell-Zabaleta, Samuel Durán-Agüero, Diana Rojas-Gómez, Jorge González-Casanova, Waldo Díaz-Vásquez, Maricarmen Chacín, Lissé Angarita Dávila

**Affiliations:** 1 Centro de Investigaciones Endocrino-Metabólicas, Escuela de Medicina, Universidad del Zulia, Maracaibo 4001, Venezuela; raquelamiracano@gmail.com (R.C.); nestorag17@gmail.com (N.G.); gabrielasanteliz29@gmail.com (R.S.); pabloduran1998@gmail.com (P.D.); 2 Facultad de Ciencias de la Salud, Universidad Simón Bolívar, Barranquilla 080002, Colombia; 3 Escuela de Medicina, Facultad de Medicina, Universidad del Zulia, Maracaibo 4001, Venezuela; arturojboscanmd@gmail.com; 4 Escuela de Nutrición y Dietética, Facultad de Ciencias para el Cuidado de la Salud, Universidad San Sebastián, Santiago 7511111, Chile; 5 Escuela de Nutrición y Dietética, Facultad de Medicina, Universidad Andres Bello, Santiago 8370321, Chile; diana.rojas@unab.cl; 6 Facultad de Ciencias de la Salud, Instituto de Ciencias Biomédicas, Universidad Autónoma de Chile, Santiago 8910060, Chile; 7 Escuela de Nutrición y Dietética, Facultad de Medicina, Universidad Andres Bello, Concepción 4260000, Chile

**Keywords:** gut microbiota, polyphenols, flavonoids, diabetes mellitus, steatotic liver disease, cardiovascular disease

## Abstract

The intricate interplay between the gut microbiota and polyphenols has emerged as a captivating frontier in understanding and potentially harnessing the therapeutic potential of these bioactive compounds. Phenolic compounds, renowned for their antioxidant, anti-inflammatory, antidiabetic, and anticancer properties, are subject to intricate transformations within the gut milieu, where the diverse microbial ecosystem exerts profound effects on their metabolism and bioavailability. Conversely, polyphenols exhibit a remarkable capacity to modulate the composition and activity of the gut microbiota, fostering a bidirectional relationship that extends beyond mere nutrient processing. This symbiotic interaction holds significant implications for human health, particularly in cardiometabolic diseases such as diabetes mellitus, metabolic-dysfunction-associated steatotic liver disease, and cardiovascular disease. Through a comprehensive exploration of molecular interactions, this narrative review elucidates the reciprocal dynamics between the gut microbiota and polyphenols, unveiling novel avenues for therapeutic intervention in cardiometabolic disorders. By unravelling the intricate cross-talk between these two entities, this review underscores the multifaceted roles of polyphenols in overall health and the pivotal role of gut microbiota modulation as a promising therapeutic strategy in mitigating the burden of cardiometabolic diseases.

## 1. Introduction

The human intestine is a complex ecosystem harboring a diverse and abundant microbial community, predominantly composed of bacteria, viruses, fungi, protozoa, and archaea, collectively known as the gut microbiota [1]. The gut microbiota has emerged as a critical determinant of health and homeostasis, gaining increasing attention from the scientific community due to the growing body of evidence linking its composition and function to various health and disease states [2].

Although it can vary among individuals due to numerous, not yet fully understood factors [3], it is known that the human intestinal flora is predominantly composed of six bacterial phyla: *Firmicutes*, *Bacteroidetes*, *Actinobacteria*, *Proteobacteria*, *Fusobacteria*, and *Verrucomicrobia*. Among these, Firmicutes and Bacteroidetes are the most abundant. Under optimal health conditions, the gut microbiota maintains remarkable stability, exhibiting resilience and effective symbiotic interactions with the host organism [2].

Current evidence suggests that diet can cause significant changes in the activity and composition of the gut microbiota. Variations in the intake of different macronutrients, such as carbohydrates, lipids, and proteins, have been associated with alterations in the gut microbiota [4]. For instance, a high-fat diet may induce dysbiosis, characterized by a reduction in the proportion of Bacteroidetes and an increase in the phyla Firmicutes and Proteobacteria. Furthermore, increased protein intake is related to a higher abundance of the genera Bacteroides, Alistipes, and Bilophila. On the other hand, a high intake of digestible carbohydrates has been shown in murine models to correlate with an increased abundance of Escherichia coli, which is associated with higher intestinal inflammation [5].

The gut microbiota plays a crucial role in the host’s defense against pathogens through an interplay with the immune system. These mechanisms include host defense against pathogens through competitive exclusion and modulation of immune cell activity, thus actively participating in the functioning of the immune system; the synthesis, absorption, and metabolism of a wide variety of nutrients; and modifying the bioavailability of diverse dietary molecules, including vitamins and certain organic compounds such as polyphenols [6,7].

A compelling link has emerged between gut microbiota alterations (or dysbiosis) and a wide spectrum of pathologies, including cardiometabolic diseases such as obesity, diabetes mellitus, non-alcoholic fatty liver disease, and cardiovascular disease [8,9]. These conditions carry a significant burden of morbidity and mortality, and their increasing prevalence poses a major public health challenge. Consequently, the active pursuit of effective preventive and therapeutic strategies for these diseases is paramount. By improving disease management and addressing the underlying mechanisms, we can mitigate the detrimental impact of these conditions on public health [10,11,12,13].

Emerging evidence suggests that polyphenol consumption can enhance human health through diverse mechanisms, potentially positioning them as novel therapeutic targets for various diseases [14,15,16,17]. Given the crucial role of the gut microbiota in nutrient metabolism and absorption and the potential of polyphenol consumption to modulate the gut microbiome, investigating the interactions between these entities is paramount. Understanding these interactions is essential for elucidating the mechanisms underlying polyphenols’ health benefits and proposing novel therapeutic applications [18].

In light of the intricate interplay between the gut microbiota and polyphenols, this narrative review aims to synthesize the current understanding of their bidirectional interactions from a molecular perspective and explore their potential as therapeutic targets for cardiometabolic disease management.

## 2. Phenolic Compounds

Phenolic compounds are naturally occurring compounds synthesized by plants that are widely distributed in plants and possess potent antioxidant properties. Based on their chemical structures, natural polyphenols are classified into four main categories: flavonoids, phenolic acids (accounting for 60% and 30% of total natural polyphenols, respectively), lignans, and stilbenes, which further comprise subcategories. Additional subclasses not included in the currently recognized ones are summarized in Table 1; this list is not exhaustive; only the most studied phenolic compounds are mentioned. Phenolic compounds are ubiquitous components of human and animal diets, being abundantly present in various plants, including fruits, vegetables, spices, whole grains, berries, teas, flowers, and more [19].

Polyphenols have long been associated with various health benefits attributed to their potent antioxidant properties. However, compelling evidence now highlights the ability of polyphenols to modulate inflammation, regulate metabolic pathways, and interact with a myriad of signaling proteins independent of their REDOX properties. This multifaceted action positions polyphenols as promising candidates for therapeutic applications in multiple diseases [20].

**Table 1:** Classification of the phenolic compounds family and their dietary sources.

Classification	Subclassification	Compounds	Dietary Sources	Refs.
Flavonoids	Flavones	Apigenin	Parsley, sorghum, chamomile, celery, spinach, artichokes, oregano	[21]
		Luteolin	Parsley, sorghum, carrots, broccoli, onion greens, chrysanthemum flowers	[22,23]
	Flavonoles	Quercetin	Citrus fruits, nuts, olive oil, sorghum, green tea, red grapes, wine, cherries, berries such as blueberries	[24,25]
		Kaempferol	Apple, grape,sorghum, tomato, green tea, broccoli, ginkgo leaves	[26]
		Myricetin	Grapes, oranges, berries, red wine	[27]
	Flavanoles	Catechins	Chocolate, green tea, apricots, nueces, barley grains	[28]
		Epigallocatechin gallate	Green tea	[29,30,31]
	Flavanones	Naringenin	Grapefruit, orange, tart cherries, tomatoes, greek oregano	[32,33]
		Hesperetin	Lemons, limes, tangerines, grapes	[34]
	Isoflavones	Puerarin	Pueraria lobata	[35]
		Genistein	Soy and soy-based foods	[36]
		Daidzein	Soy and soy-based foods	[37]
	Anthocyanins	Cyanidin	Apples, plums, berries, red onions	[38]
		Pelargonidin	Berries, strawberries, blueberries, red radishes, blackberries	[39]
		Delphinidin	Berries, eggplants, roselle, wine	[40]
Phenolic acid	Hydroxybenzoic acid	Gallic acid	Guava leaves,sorghum, rye, pomegranate root bark, chestnuts, tea leaves	[23,41]
	Hydrocinnamic acid	Ferulic acid	Rice, wheat,sorghum, rye, oats, pineapple, spinach, parsley, rye, barley	[42]
		Caffeic acid	Coffee, wine, tea,sorghum, rye, wheat.	[23,43]
Stilbenes		Resveratrol	Peanuts, grapes, cranberries, raspberries, blackberries, red wine	[44,45]
Lignans		Secoisolariciresinol	Flaxseed, plumes, green tea	[46]

## 3. Role of Microbiota in Absorption and Bioavailability of Phenolic Compounds

Bioavailability is the percentage of an ingested nutrient available for use and/or storage in the body. The bioavailability and, consequently, the systemic effect exerted by a micronutrient can be affected by various factors. Phenolic compounds are not exempt from variations in their bioavailability, as their absorption depends on their class, food matrix components, enzymatic metabolism in the gastrointestinal tract by human enzymes [47], and metabolism by the gut microbiota [48].

The gut microbiota is mainly composed of bacteria from the phyla *Bacteroidetes*, *Firmicutes*, *Actinobacteria*, and *Proteobacteria* to a lesser extent. Due to multiple endogenous and exogenous factors, the microbiota composition can change, leading to dysbiosis in some cases. These highly varied factors include diet, drug administration, the immune system, and gastrointestinal diseases. [49]. Interindividual variability in the gut microbiota may be, at least in part, the reason why the bioavailability of phenolic compounds can vary from person to person. Thus, there are specific bacterial groups with the ability to biotransform certain polyphenols in particular, among which are *Flavonifractor plautii*, *Slackia equolifaciens*, *Slackia isoflavoniconvertens*, *Adlercreutzia equolifaciens*, *Eubacterium ramulus*, *Eggerthella lenta*, and *Bifidobacterium* spp. [18]. Therefore, it is crucial to examine how the microbiota influences the metabolism of medically relevant polyphenols to understand its role in the potential pharmacological effects of these promising compounds. The following sections describe the impact of the microbiota on the metabolism of several clinically significant phenolic compounds, focusing on those most extensively studied. However, it is important to note that numerous other compounds may not be included in this overview.

### 3.1. Quercetin

Quercetin (3,5,7,3′,4′-pentahydroxyflavone) is a flavonoid found in plant-based foods such as apples, dill, asparagus, cilantro, celery, onions, wine, vegetables, tea, and capers. It has demonstrated multiple pharmacological properties [50] and is being studied for its potential benefits in cardiovascular disease and non-alcoholic fatty liver disease [51]. Quercetin derivatives are deconjugated and converted into hydroxyphenylacetic acids by the action of the intestinal flora, transforming these flavonoids into phenolics of a lower molecular weight, which can determine the absorption of these compounds [52]. Among the intestinal tract bacteria metabolizing quercetin are *Clostridium orbiscindens* and *Eubacterium ramulus* [53,54,55,56].

Preclinical evidence suggests that the bioavailability of quercetin and its active metabolites may vary among individuals, depending on bacterial degradation. In mice associated with the human microbiota, changes in the concentrations of the metabolites were correlated with the presence of different bacterial groups; specifically, a positive correlation was observed between the ratio of Log10 Enterobacteriaceae count/Log10 *Clostridium coccoides* group count and the plasma concentrations of quercetin metabolites (quercetin and isorhamnetin) (*p* = 0.008). Additionally, a negative correlation was found between the ratio of Log10 *Clostridium leptum* subgroup count/Log10 Enterobacteriaceae count and the plasma concentrations of quercetin metabolites (*p* = 0.005). Both of these results were statistically significant. On the other hand, there was a weak positive correlation with the Bifidobacterium group (*p* = 0.096), but this result was not statistically significant [57].

On the other hand, quercetin is commonly found in the form of rutin glycoside, which is not easily absorbed. Therefore, bacterial degradation is crucial for its effectiveness. In in vitro porcine ileal and colonic fermentation models, it was observed that the colonic degradation rate was higher than the ileal rate, and in turn, the appearance of quercetin was correlated with a higher abundance of *Firmicutes* and varied according to the ileal inoculum, suggesting that the absorption of this flavonoid can be improved by modifications of the microbiota [58].

### 3.2. Resveratrol

Resveratrol (3,5,4′-trihydroxy-trans-stilbene) is a stilbenoid polyphenol found in 70 plant species, with high concentrations in grapes, peanuts, blackberries, and, to a lesser extent, in red wines [59]. It has potential benefits in treating diabetes mellitus, metabolic syndrome, and cardiovascular disease. However, resveratrol in plasma and tissues after oral administration is barely detectable, highlighting the importance of its metabolites as the main contributors to its health effects [60]. In this regard, a study comparing the biotransformation of resveratrol in groups of mice with different microbiota compositions found differences in the concentrations of metabolites such as resveratrol sulfate (RES-sulfate), resveratrol glucuronide (RES-glucuronide), and dihydro resveratrol (DHR), which were significantly increased in mice colonized by *Ligilactobacillus salivarious* (Li01) compared to germ-free mice. Similarly, the metabolism of resveratrol was compared in mouse models with sodium dextran sulfate-induced colitis and conventional mice, showing variations in its metabolism. In the presence of dysbiosis, there was an increase in the concentration of resveratrol in feces, indicating a decrease in its catabolism. However, this increase decreased after 29 days of resveratrol treatment, possibly due to its ability to promote the recovery of normal flora [61].

In a study by Li et al. [62], eleven resveratrol metabolites were identified following oral administration over 4 weeks in a mouse model. These metabolites included DHR, Lunularin (LUN), three resveratrol conjugates (RES-sulfate, RES-glucuronide, and resveratrol sulfoglucuronide), four DHR conjugates (DHR-sulfate, DHR-glucuronide, DHR-biglucuronides, and DHR-sulfate-glucuronide), and two LUN conjugates (LUN-sulfate and LUN-glucuronide). The study demonstrated that resveratrol underwent extensive metabolism, resulting in very low or undetectable levels in tissues and body fluids, while its metabolites, particularly DHR, LUN, and their conjugates, were found at higher concentrations. Furthermore, DHR and LUN were shown to be microbiota-derived metabolites, as both their conjugates disappeared in mice treated with antibiotics for 5 days [62].

Regarding the clinical evidence, Iglesias et al., in a study involving 195 healthy individuals, revealed that the gut microbiota can modulate the response to resveratrol treatment through various metabotypes. It was observed that 74% of participants were LUN producers, while 26% were not. This difference suggests that the availability of LUN could explain the variations in the effects of resveratrol. Additionally, a statistically significant association was found between LUN production and female sex (*p* < 0.05) but not with age or body mass index (BMI). The authors proposed that resveratrol is converted to DHR and, depending on the gut microbiota’s ability to produce LUN, DHR is sequentially dehydroxylated at position 5 to form LUN and at position 3 to produce 4-hydroxybenzyl alcohol. In contrast, individuals who are not LUN producers cannot dehydroxylate at positions 3 and 5 [63].

### 3.3. Catechins

Catechins (3,3′,4′,5,7-pentahydroxyflavan) and their derivatives are found in various foods and herbs, including tea, apples, persimmons, cocoa, grapes, and berries. They are particularly abundant in green tea, which is rich in polyphenolic compounds such as (-)-epigallocatechin gallate (EGCG), (-)-epigallocatechin (EGC), 3-gallate of (-)-epicatechin (ECG), and (-)-epicatechin (EC) [64]. These compounds have been linked to various health benefits, including protection against cancer, obesity, diabetes, and cardiovascular diseases. They also exhibit antioxidant, hepatoprotective, and neuroprotective properties [65,66,67,68].

Extensive preclinical evidence suggests that the degradation of EC, EGC, ECG, and EGCG by the gut microbiota happens rapidly, with these compounds remaining unchanged for only a brief period after reaching the colon [69,70]. EGCG is particularly susceptible to rapid breakdown through degallation, C-ring opening, and A-ring fission, generating metabolites like diphenylpropanols, phenylvalerolactones, and phenylvaleric acids. These metabolites undergo further reduction in their aliphatic chain length and dehydroxylation of the phenyl group, leading to a series of hydroxylated phenylcarboxylic acids. Specific bacteria have been implicated in the aforementioned catabolic processes. For instance, C-ring breakage can be mediated by microorganisms such as *Lactobacillus plantarum*, *Eggerthella lenta*, *Flavonifractor plautii*, and the genus *Eggerthella*. The metabolites have also been correlated with distinct bacterial taxa [69].

Liu et al. identified 12 metabolites resulting from the degradation of EC by the human gut microbiota. These metabolites include 3,4-diHPP-2-ol, 3-HPP-2-ol, 4H-diHPVA, 3,4-diHPV, 4H-HPVA, 3-HPV, 3,4-diHPVA, 3-HPVA, 3,4-diHPPA, 3-HPPA, 3-HPAA, and 4-HPAA. The study also revealed marked inter-individual differences in the concentration and types of EC metabolites produced and the metabolic rate. Statistically significant correlations were found for 3-HPP-2-ol, including a negative correlation with the abundance of *Bacteroidetes* (*p* < 0.05) and positive correlations with the abundance of *Firmicutes* (*p* < 0.05), *Verrucomicrobia* (*p* < 0.01), *Actinobacteria* (*p* < 0.05), and *Tenericutes* (*p* < 0.01). Additionally, a statistically significant positive correlation was identified for 3,4-diHPP-2-ol with *Actinobacteria* (*p* < 0.05); for 3-HPV with *Verrucomicrobia* (*p* < 0.05); for 3-HPVA with *Tenericutes* (*p* < 0.01) and *Fibrobacteres* (*p* < 0.05); and for 3-HPPA with *Tenericutes* (*p* < 0.01) [71,72]. The bacterial metabolism of (+)-catechin exhibited variations between individuals and among different colon regions within the same individual [73].

### 3.4. Chlorogenic Acid

Chlorogenic acid (5-O-caffeoylquinic acid) is a phenolic compound found in various foods and herbs, including apples, coffee beans, tea, and tomatoes. This polyphenol offers numerous health benefits, including antioxidant, anti-inflammatory, and antidiabetic properties [74]. In a study by Mortelé et al., fecal samples from obese women (n = 4) and lean women (n = 9) were analyzed. Significant differences in microbiota composition were observed between the two groups. Specifically, obese women showed fewer anaerobic bacteria than lean women.

Additionally, a decrease in microbiota metabolic activity was found in the obese group, resulting in lower biotransformation of chlorogenic acid. A notable disparity in the variety of metabolites present in both groups was identified, with 23 biotransformation products identified in the lean population and 13 in the obese population [75], demonstrating the crucial role of the microbiota in the metabolism of this polyphenol. Caffeic acid (3′,4′-dihydroxycinnamic acid), mainly synthesized through the hydrolysis of chlorogenic acid in the colon, also exhibits inter-individual variations in metabolite production by the gut microbiota. The main metabolites generated include 3-(3′,4′-dihydroxyphenyl)propanoic acid (also known as dihydrocaffeic acid), 3-(3′-hydroxyphenyl)propanoic acid, and phenylacetic acid [76].

### 3.5. Curcuminoides

Curcuminoids, bioactive polyphenolic compounds found in turmeric, include curcumin (1,7-bis(4-hydroxy-3-methoxyphenol)-1,6-heptadiene-3,5-dione), demethoxycurcumin, and bisdemethoxycurcumin. Curcumin, in particular, has attracted significant attention for its anti-inflammatory, antidiabetic, anticancer, and anti-ageing properties. However, its effectiveness in medical treatments is hindered by its inability to dissolve well in water, inefficient absorption and distribution in the body, and poor bioavailability [77]. Preclinical evidence suggests that the human gut microbiota metabolizes curcuminoids. In an in vitro model using human fecal matter, it was observed that within 24 h, 24% of curcumin, 61% of demethoxycurcumin, and 87% of bisdemethoxycurcumin were degraded. The main metabolites detected in this biotransformation were tetrahydrocurcumin, dihydroferulic acid, and a metabolite provisionally identified as 1-(4-hydroxy-3-methoxyphenyl)-2-propanol [78].

Various formulations have been developed to enhance the absorption and bioavailability of curcuminoids, optimizing their absorption in the small intestine and altering their biotransformation by the gut microbiota. A preclinical study using in vitro models demonstrated that a lecithin–curcuminoid extract increases the degradation of curcuminoids compared to an unformulated extract. After 24 h of fecal fermentation, curcumin was degraded by 22.7% in the unformulated extract, whereas the degradation reached 63.5% in the formulated extract [79]. Regarding specific bacteria capable of biotransforming curcumin, several have been identified, including *Bifidobacterium longum*, *Bifidobacterium pseudocatenulatum*, *Enterococcus faecalis*, *Lactobacillus acidophilus*, *Lactobacillus casei*, and *Escherichia coli*. The latter utilizes an NADPH-dependent curcumin/dihydrocurcumin reductase enzyme to convert curcumin into dihydrocurcumin, which is subsequently transformed into tetrahydrocurcumin [80].

A preclinical study conducted in vivo with murine models revealed the production of specific curcumin metabolites exclusively by the gut microbiota. Notably, these include hexahydro-dimetylcurcumin and hexahydro-didemetylcurcumin, which were undetectable in the presence of antibiotics that disrupt intestinal flora. This finding underscores the critical role of gut bacteria in curcumin metabolism. Furthermore, the study demonstrated that the gut microbiota can convert certain curcumin metabolites back into their bioactive forms through deconjugation processes. This phenomena highlights the complex interaction between curcumin and the gut microbiota and suggests that microbiota-mediated transformations are crucial for the therapeutic efficacy of curcumin [81].

## 4. Impact of Phenolic Compounds on the Microbiota

**Figure 1:**
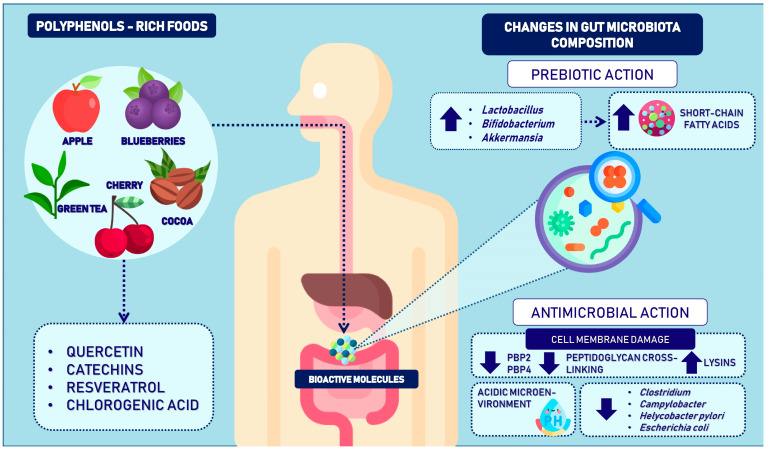
Impact of phenolic compounds on gut microbiota composition and function. Phenolic compounds are bioactive molecules capable of inducing numerous beneficial effects in our organism by modulating the gut microbiota composition. These compounds exert a positive modulation through two main mechanisms: 1. A prebiotic effect, promoting the growth and production of beneficial bacteria and consequently increasing the levels of short-chain fatty acids. 2. An antimicrobial effect, suppressing pathogenic bacteria by altering their structural and functional integrity through the inhibition of PBP2 and PBP4, leading to reduced peptidoglycan cross-linking and increased lysine content as well as creating an acidic microenvironment by proton donation, impairment of proton pumps, and depletion of H+-ATPase5.

Among the diverse functions associated with phenolic compounds, the influence of these bioactive molecules on the composition of the gut microbiota has gained significant research attention [82], highlighting the intricate bidirectional relationship between polyphenols and the microbiota, offering an explanation for the antioxidant, anti-inflammatory, antidiabetic, and anticancer properties attributed to polyphenols through their positive modulation of the gut microbiome [83]. In this regard, polyphenols have been ascribed to prebiotic activity for enhancing gut microbiota composition (Figure 1). However, the underlying mechanisms remain unclear.

Hypotheses proposed to explain these mechanisms postulate direct and indirect interventions in bacterial growth. Polyphenols can exert antimicrobial activity against pathogenic bacteria while promoting the growth of commensal bacteria beneficial to the host [84]. Evidence from clinical and preclinical studies indicates that specific doses of polyphenols can modify the microbiota, inhibiting certain groups of bacteria and enhancing the growth of others [85]. In preclinical studies, Cladis et al., in mouse models, observed dose-dependent changes in the gut microbiota following the administration of blueberry polyphenols, increasing diversity to a healthier gut microbiota at moderate doses but decreasing it at high doses [86]. Similarly, another study in mouse models with antibiotic-induced gut dysbiosis evaluated the effects of tea polyphenols. It demonstrated a regulatory effect on the diversity of the microbiota composition following antibiotic treatment, regulating the increase in *Firmicutes* and the decrease in *Bacteroidetes* [87].

In a mouse model of high-fat-diet-induced obesity, resveratrol administration was shown to suppress obesity-associated gut microbiota, including *Lactococcus*, *Clostridium XI*, *Oscillibacter*, and *Hydrogenoanaerobacterium*. This suppression was mediated by inhibiting mTORC1, a key regulator of energy metabolism, by activating mTOR complex 2 signaling [88]. Additionally, other studies have demonstrated that resveratrol increases the abundance of *Lactobacillus*, *Bifidobacterium*, and *Akkermansia muciniphila*, bacterial taxa associated with weight loss, intestinal barrier protection, and reduced inflammation [89].

Furthermore, curcumin administration in animal models has reduced the abundance of more than 36 potentially harmful bacterial strains [90], including several genera associated with diabetes and inflammation, such as *Ruminococcus* [91]. In line with these findings, a study by Shen et al. demonstrated that a 15-day treatment with 100 mg/kg body weight of curcumin in mice significantly altered the gut microbiota composition, increasing the abundance of Prevotellaceae, Bacteroidaceae, and Rikenellaceae [91].

Meanwhile, an in vitro study using human fecal samples by Tzounis et al. [92] demonstrated the positive influence of flavonoids on the growth of *Bifidobacterium* and *Lactobacillus*. They incubated epicatechin and catechin (150 mg/L or 1000 mg/L) with fecal bacteria and observed a statistically significant increase in *Clostridium coccoides-Eubacterium rectale*, *Bifidobacterium* spp., and *Escherichia coli*. Additionally, there was a decrease in *C. histolyticum* growth. In the case of epicatechin, a significant increase in the growth of the *C. coccoides-Eubacterium rectale* group was observed [92]. Similarly, a prebiotic effect has been observed for berry polyphenols, which stimulate the beneficial growth of bacteria such as *Bifidobacterium*, *Lactobacillus*, and *Akkermansia* [93].

In a randomized, placebo-controlled clinical trial by Peterson et al., either curcumin tablets, curcumin, or placebo were administered. The study revealed significant and individualized changes in gut microbiota composition over time, with a marked increase in those treated with curcumin compared to the control group [94], supporting the protective effects of curcumin through its ability to shift the balance from pathogenic to beneficial bacteria in the intestine.

A randomized, crossover, controlled intervention trial involving 10 men aged 45–50 years investigated the effects of red wine consumption on gut microbiota and health parameters. Participants consumed red wine, rich in anthocyanins, flavan-3-ols, type B proanthocyanidins, flavonols, stilbenes, and phenolic acids, for 4 weeks. This intervention significantly increased the abundance of bacterial taxa belonging to the *Enterococcus*, *Prevotella*, *Bacteroides*, *Bifidobacterium*, *Bacteroides uniformis*, *Eggerthella lenta*, and *Blautia coccoides-Eubacterium rectale* groups. Concurrently, blood pressure, cholesterol, triglycerides, and C-reactive protein were decreased [95]. In line with the findings presented, studies by Zorraquin et al. [96] and Dueñas et al. [97] further substantiate the beneficial effects of red wine consumption on gut microbiota composition [96,97]. These studies explored the interplay between red wine consumption and the gut microbiota and observed an increase in the abundance of bacteria beneficial to the host. However, both studies emphasize the need for further research.

A dietary intervention study investigating the effects of cherry consumption on the gut microbiota revealed alterations in the microbial composition of the participants. Cherries, rich in anthocyanins, flavonoids, chlorogenic acid, and neochlorogenic acid, were administered to the participants. However, the changes in gut microbiota composition depended on the individuals’ initial microbial composition. In participants with a high abundance of *Bacteroides*, cherry consumption led to a decrease in *Bifidobacterium* and an increase in *Firmicutes* and *Collinsella*. Conversely, in the group with a low initial *Bacteroides* abundance, cherry consumption increased *Bacteroides*, *Prevotella*, and *Bifidobacterium*, accompanied by a decrease in *Lachnospiraceae*, *Ruminococcus*, and *Collinsella* [98]. Duda-Chodak et al. investigated the influence of polyphenols, including naringenin, hesperetin, quercetin, rutin, and catechin, on the growth of various gut microbiota bacteria, such as *Bacteroides galacturonicus*, *Lactobacillus sp*., *Enterococcus caccae*, *Bifidobacterium catenulatum*, *Ruminococcus gauvreauii*, and *Escherichia coli* [99]. The study found that naringenin and hesperetin aglycones inhibited the growth of all analyzed bacteria.

Similarly, Stapleton et al. demonstrated that when co-administered with methicillin-resistant Staphylococcus aureus (MRSA), epicatechin gallate sensitized the bacteria to the action of β-lactam antibiotics [100]. This sensitization was attributed to reduced PBP2 and PBP4 activities, leading to decreased peptidoglycan cross-linking in the cell wall and increased autolysin activity. These findings suggest that polyphenols may interact with bacteria to reduce their growth through mechanisms involving membrane structure and function disruption, leading to increased permeability and sensitization to the antibacterial action of drugs [82].

Phenolic extract mixtures have been shown to enhance the inhibition of *Helicobacter pylori*, a bacterium associated with gastric ulcers and inflammation. This enhanced inhibitory effect is attributed to a membrane damage mechanism involving the creation of an acidic microenvironment through proton donation, deterioration of proton pumps, and loss of H+-ATPase activity. These alterations disrupt cellular function and ultimately lead to bacterial death [101].

Emerging scientific evidence has ascribed prebiotic properties to polyphenols, emphasizing their ability to promote the growth of beneficial gut bacteria such as *Lactobacillus*, *Bifidobacterium*, and *Akkermansia*. These beneficial bacteria contribute to increased short-chain fatty acids (SCFAs) and overall host health [102]. Additionally, polyphenols exhibit antibacterial activity by suppressing the growth of pathogenic bacteria, including *Clostridium*, *Staphylococcus*, *Enterococcus*, *Campylobacter*, *H. pylori*, and *Escherichia coli*, through damage to their cell membranes [103].

Polyphenols have emerged as promising agents for counteracting various cardiometabolic diseases, including lipid metabolism dysregulation, insulin resistance, inflammation, and oxidative stress development (Table 2). Upon reaching the large intestine, polyphenols are transformed by the colonic microbiota, as they are not fully digested in the gastrointestinal tract and are therefore considered prebiotic molecules [104].

### 4.1. Diabetes Mellitus

Numerous animal and human studies have demonstrated the broad beneficial effects of phenolic compounds, including quercetin, resveratrol, epicatechin, and chlorogenic acid (CGA), on diabetes mellitus (DM) [108,110,111]. Phenolic compounds have positively affected hyperglycemia, hyperinsulinemia, and insulin resistance. Additionally, they have been shown to possess systemic antioxidant and anti-inflammatory properties and to promote gut microbiota health, which may be beneficial for preventing and treating diabetes and its complications (Table 3) [83,105,112].

It is increasingly recognized that the gut microbiota may contribute to diabetic complications, including diabetic nephropathy (DN). Disruption of the gut microbiota and its metabolites has been associated with DN development, and those mechanisms have been studied in preclinical studies [119]. Short-chain fatty acids (SCFAs), produced by gut bacteria, have demonstrated the ability to mitigate the progression of diabetic complications [107]. This was evident in preclinical studies in db/db mice fed a diet with or without 0.4% resveratrol for 12 weeks. Resveratrol supplementation appears to have improved DN progression and alleviated tubulointerstitial fibrosis. Further preclinical studies may have revealed that resveratrol may modulate gut microbiota dysbiosis, characterized by the expansion of SCFA-producing bacteria such as *Faecalibaculum* and *Lactobacillus*, leading to increased SCFA concentrations (particularly acetic acid) in feces [120,121]. These findings suggest that resveratrol-mediated alterations in the gut microbiome may play a crucial role in its mechanism of action, supporting the gut–kidney axis in DN.

In this context, the mechanism for improving glycemic tolerance has been linked to CGA’s ability to reduce triglyceride (TG) synthesis and fatty acid transport, possibly by stimulating hepatic lipolysis and gluconeogenesis, leading to a decrease in the expression of genes involved in TG synthesis and fatty acid transport [107]. This protective mechanism of CGA against hyperglycemia has emerged as a potential therapeutic target, prompting Yang et al. [107] to investigate its efficacy by administering CGA or metformin systemically to db/db diabetic mice. Their findings demonstrated that exogenous CGA administration may reduce the hepatic lipid content by upregulating the expression of CPT1a (carnitine palmitoyltransferase 1a), ACOX1 (acyl-CoA oxidase 1), ATGL (adipose triglyceride lipase), and HSL (hormone-sensitive lipase). In contrast, these antilipidemic effects are promising. Further exploration of the signaling pathways that regulate lipid metabolism is warranted, including the effects of CGA at earlier stages of the disease and different concentrations.

Furthermore, phenolic compounds have been shown to protect against oxidative stress, a major contributor to diabetes and its complications. In this context, preclinical studies have investigated the ability of phenolic compounds, such as CGA, to restore the expression of inflammatory genes, including TNF-α (tumor necrosis factor-alpha), IL-1β (interleukin-1beta), IL-6, and IL-10, as well as genes encoding antioxidant enzymes, including SOD1 (superoxide dismutase 1), SOD2 (superoxide dismutase 2), and GPX1 (glutathione peroxidase-1) in db/db diabetic mice [107]. Similarly, preclinical studies suggest that quercetin may have a protective effect against peripheral diabetic neuropathy in rats [121] by improving gut health and reducing oxidative stress. Specifically, quercetin administration in rats with streptozotocin-induced peripheral diabetic neuropathy may increase the abundance of beneficial bacteria, such as Lactobacillus and *Bifidobacterium*, while decreasing the abundance of pathogenic bacteria such as *E. coli* and *Clostridium perfringens* and also reducing levels of reactive oxygen species in the gut [122].

On the other hand, clinical studies have demonstrated that phenolic compounds such as CGA may restore the abundance of Lactobacillus, a bacterial population well known for stimulating the production of insulinotropic polypeptides and GLP-1 (glucagon-like peptide-1), which translates to increased glucose uptake by muscles and enhanced stimulation of hepatic glucose uptake from the bloodstream. Antidiabetic effects have been demonstrated after eight weeks of *Lactobacillus casei* supplementation in forty diabetic patients, who achieved an improved glycemic response, increased SIRT1 expression, and decreased fetuin-A levels compared to the placebo group. Additionally, anthropometric parameters, serum insulin levels, and the HOMA-IR index were improved [123]. However, further elucidation is warranted regarding the microbial changes contributing to the beneficial effects of CGA, particularly its hypolipidemic effects.

Dietary intervention with functional foods rich in phenolic compounds, soluble fiber, high-quality plant proteins, and a low glycemic index may benefit fecal microbiota composition, providing potential therapies for improving glycemic control, dyslipidemia, and inflammation in individuals with type 2 diabetes. Additionally, a significant increase in serum antioxidant activity was observed in the functional food intervention group compared to the placebo group. Therefore, it is suggested that polyphenols may benefit type 2 diabetes through dietary intervention with functional foods [124].

In another line of clinical research, a dietary portfolio (DP) consisting of functional foods rich in phenolic compounds, soluble fiber, high-quality plant proteins, and a low glycemic index was also investigated for its beneficial effects. The foods included in the DP were inulin, chia seeds, soy protein, dehydrated cactus pear (for its prebiotic properties), omega-3 fatty acids, plant protein, polyphenols, and soluble and insoluble fiber, all of which are known to restore the gut microbiota and attenuate biochemical abnormalities and metabolic endotoxemia caused by microbiota dysbiosis in patients with type 2 diabetes [124].

The systematic review with a meta-analysis by Raina et al. (2024) underscores the therapeutic potential of phenolic compounds in diabetes mellitus. Exploring their mechanisms of action reveals their antioxidative, anti-inflammatory, and antidiabetic capabilities, offering a promising strategy to combat diabetic complications. Furthermore, it suggests the inclusion of phenolic compounds in the diet as a long-term approach for diabetes management [17].

### 4.2. Metabolic-Dysfunction-Associated Steatotic Liver Disease (MASLD)

Currently, no effective treatment is available for metabolic-dysfunction-associated steatotic liver disease (MASLD), previously known as nfon-alcoholic atty liver disease (NAFLD) [125]. Therefore, the discovery of specific therapeutic strategies based on its pathogenesis is of paramount importance. The most studied polyphenols in MASLD include quercetin, resveratrol, green tea flavonoids, soy isoflavones, and rutin, along with silymarin, both in animal models and humans [104,113,115,126]. Probiotics can alleviate MASLD progression by activating the AMPK pathway to phosphorylate ACC, blocking the SREBP-1/Fas signaling pathway, inhibiting lipogenesis, and increasing fatty acid oxidation [127,128,129,130].

High-fat-diet-induced obesity, glucose and lipid metabolism disorders, and MASLD are often accompanied by an increased abundance of *Firmicutes* and a decreased abundance of *Bacteroidetes* and *Proteobacteria* [107,131]. Recent studies, both preclinical [109,121,122,128,131,132] and clinical [112,113,115,119,124,126], have shown that phenolic compounds such as curcumin, quercetin, isoquercetin, and rutin can modulate the abundance of Rikenellaceae, Bacteroidaceae, and Prevotellaceae in the gut microbiota and improve metabolic health parameters such as dyslipidemia, hyperglycemia, and obesity. However, there are still contradictory data, and more studies are needed to determine their potential role in treating MASLD [113,124].

Nonetheless, preclinical evidence suggests that CGA may increase the Bifidobacterium content and reduce *Escherichia coli* in MASLD mice [106] and inhibit the growth of *Desulfovibrionaceae*, *Ruminococcaceae*, *Lachnospiraceae*, and *Erysipelotrichaceae* while increasing the growth of *Bacteroidaceae* and *Lactobacillaceae*. These interactions may play a beneficial role in liver metabolism and the reduction in fat accumulation in the liver in conditions such as MASLD.

Furthermore, fecal microbiota transplant (FMT) experiments have demonstrated that *Enterococcus faecalis* causes liver inflammation and fat deposition along with a decrease in carnitine palmitoyltransferase-1 alpha (CPT1α) expression, suggesting that targeting *Enterococcus faecalis* with polyphenols like protocatechuic acid (PCA) may be a promising therapeutic approach. Gut microbiota characterization revealed that PCA primarily reduced the Firmicutes/Bacteroidetes ratio by decreasing *Enterococcus* abundance, which was positively correlated with c-LDL, AST, and ALT levels and most positively regulated liver lipids [133].

On the other hand, resveratrol and luteolin have been shown to interact with gut-derived hormones such as ghrelin and GLP-1 to influence liver metabolism and reduce hepatic fat accumulation. These polyphenols can lower ghrelin levels by binding to its receptor GHS-R1a, activating the AMPK pathway, and subsequently inhibiting hepatic glucose production [106].

Similarly, it has been observed that gut dysbiosis can lead to an imbalance of bile acids and choline, microbial translocation, and endotoxemia. Lipopolysaccharides produced by microorganisms can increase liver fat and inflammation by activating the inflammasome pathway [127]. One of the signaling pathways studied for inhibiting MASLD has been the use of resveratrol and luteolin on bile acid (BA) metabolism and TGR5/FXR receptors, which may contribute to the inhibition of MASLD. Tea polyphenols can decrease the action of bile salt hydrolase in the intestine and increase FXR signaling to regulate CYP7B1 activity in the liver, decreasing cholesterol synthesis [127]. Additionally, polyphenols can improve gut microbiota dysbiosis and reduce liver inflammation by promoting SCFAs, helping to mitigate inflammation and gluconeogenesis in the liver [134].

Additionally, polyphenols can stimulate GLP-1 release from the intestinal epithelium, leading to increased fatty acid oxidation, suppression of de novo lipogenesis, and reduced glucose production through gut–brain–liver communication [134].

Therefore, a combination of polyphenols and pharmaceuticals may help to alleviate and treat MASLD by influencing these metabolic pathways that also involve gut microorganisms, enhancing communication between the neuroregulatory organs of the brain.

### 4.3. Mechanisms for Combination Therapies in MASLD

**Figure 2:**
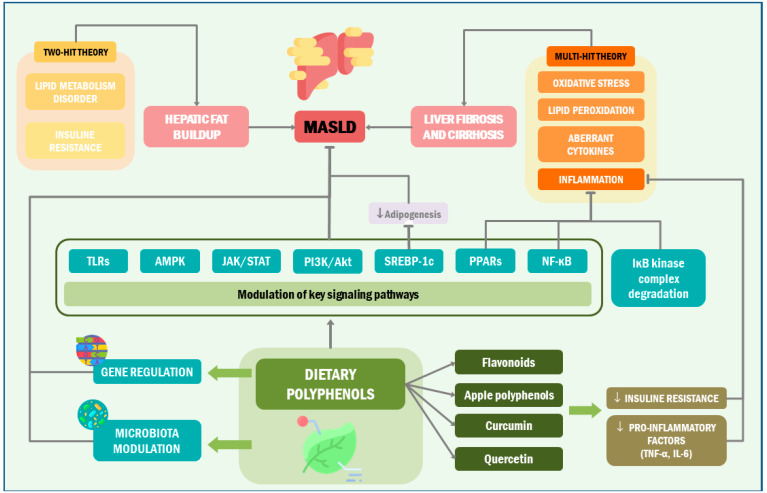
Therapeutic targets in MASLD. This illustration highlights the role of phenolic compounds in addressing metabolic-associated fatty liver disease (MASLD). These compounds modulate key pathways, including NF-κB, AMPK, and PPARs, to reduce lipid accumulation, insulin resistance, oxidative stress, and inflammation. By activating PPARs, they offer an alternative to conventional treatments. Phenolic compounds target diverse signaling pathways, providing therapeutic potential within the cardiometabolic context and reshaping MASLD management. TLRs: toll-like receptor; AMPK: AMP-activated protein kinase; JAK/STAT: Janus kinase/signal transducers and activators of transcription; PI3K/Akt: phosphatidylinositol 3-kinase (PI3K)/protein kinase B (AKT); SREBP-1c: sterol regulatory element binding protein-1c; PPARs: peroxisome proliferator-activated receptors; NF-κB: nuclear factor kappa-light-chain-enhancer of activated B cells; TNF-α: tumor necrosis factor alpha; IL-6: interleukin-6.

Research into the pathogenesis of MASLD has spurred curiosity, with its intricate mechanisms lacking definitive conclusions. The classic “two-hit theory” highlights lipid metabolism disorder and insulin resistance, fostering hepatic fat buildup [135]. Evolving into the “multiple-hit” theory, oxidative stress, lipid peroxidation, and aberrant cytokines provoke inflammation, fostering liver fibrosis and cirrhosis [136]. Key factors encompass lipid accumulation, insulin resistance, oxidative stress, and inflammatory response intertwined with diverse signaling pathways. Phenolic compounds offer promise by modulating key signaling pathways such as NF-κB, AMPK, JAK/STAT, PPARs, SREBP-1c, PI3K/Akt, and TLRs [137,138]. By targeting these pathways, phenolic compounds may mitigate lipid accumulation, insulin resistance, oxidative stress, and inflammatory responses, thus presenting a compelling avenue for MASLD prevention and treatment within the cardiometabolic context (Figure 2).

In preclinical studies, phenolic compounds combat inflammation by inhibiting the NF-κB pathway and IκB kinase complex degradation. Flavonoids, apple polyphenols, curcumin, and quercetin may reduce insulin resistance and pro-inflammatory factors TNF-α and IL-6 [139]. Transcriptomic and metabolomic preclinical studies show that polyphenolic extracts may regulate genes and pathways linked to MASLD, suppressing pro-inflammatory pathways while activating the anti-inflammatory PPAR pathway, suggesting their therapeutic potential in MASLD treatment [139].

On the other hand, PPAR receptor activation enhances β-fatty acid oxidation, which is vital for lipid metabolism and insulin sensitivity. In preclinical studies, phenolic compounds like kaempferol stimulate PPAR-α, reducing fat accumulation [140]. Tea polyphenols in MASLD mice upregulate PPARs and inhibit NF-κB, mitigating inflammation and liver fat. PPAR isoforms regulate adipogenesis, energy metabolism, and insulin sensitivity, offering therapeutic targets for metabolic disorders [141]. For instance, compounds such as kaempferol, naringenin, and glabridin have been shown to enhance the gene expression of PPAR-α, thereby mitigating obesity induced by a high-fat diet and attenuating fat deposition [142]. The administration of phenolic compounds presents an advantage in activating PPARs compared to conventional medications like Thiazolidinediones [127].

Furthermore, activation of AMPK inhibits adipogenesis by downregulating SREBP-1c, a key regulator of fat synthesis. Phenolic compounds like resveratrol, chlorogenic acid, caffeic acid, dihydromyricetin, quercetin, catechins, and curcumin may activate AMPK, reducing SREBP-1c expression, offering the potential for MASLD prevention and treatment [143]. AMPK activation also regulates the PGC-1α and SIRT-3 pathways, crucial for metabolic homeostasis [144].

### 4.4. Cardiovascular Disease

On the other hand, some phenolic compounds, such as resveratrol and flavonols, have been studied for their probable beneficial cardiovascular and anti-inflammatory mechanisms in preclinical and clinical studies [51,145,146]. In this regard, phenolic compounds may reduce inflammation and oxidative stress, preventing plaque formation in arteries and reducing the risk of cardiovascular diseases in preclinical studies. On the other hand, the gastrointestinal tract has been considered a target tissue for producing circulating inflammation with an HFD, leading to increased intestinal permeability and inflammation [147,148,149]. Based on this premise, resveratrol (RSV) studies have shown that RSV supplementation could suppress FITC-dextran absorption and decrease serum levels of LPS, TNF-1α, IL-6, IL-2, and IL-2 in preclinical studies. Moreover, RSV supplementation could also decrease MCP-1 expression in the jejunum and white adipose tissue of HFD mice. Such results could indicate an improvement in the intestinal barrier and systemic inflammation [109,149].

Furthermore, the beneficial effects of RSV on systemic inflammation and intestinal barrier integrity may be mediated by an improved gut microbiota composition. Notably, RSV has been shown to enhance the composition of gut bacteria by regulating the abundance of *Allobaculum*, *Bacteroides*, and *Blautia* genera, which are SCFA producers and negatively correlate with inflammation, insulin resistance, obesity, and other metabolic disorders in preclinical studies [150]. *Desulfovibrio*, which commonly increases after a high-fat diet, can be reduced through a probiotic-enriched dietary intervention. *Parabacteroides* are commensal bacteria that are highly effective in the gut.

Quercetin, a flavonoid polyphenol, also exhibits protective effects against cardiovascular diseases. It has been demonstrated in preclinical studies that it induces vasodilation in isolated rat arteries. In laboratory studies using hypertensive rat models, quercetin has shown the ability to reduce the severity of high blood pressure. These models include rats fed a high-sucrose diet and salt-sensitive angiotensin-induced hypertensive rats. Similar findings have been reported in a clinical trial, which showed that supplementation with epigallocatechin-3-gallate and resveratrol (EGCG+RES) for 12 weeks may affect gut microbiota composition in men but not in women and that basal microbiota composition determined increased fat oxidation after EGCG+RES supplementation [151].

The collective evidence presented highlights the potential of RSV and RSV-induced gut microbiota modulation as novel therapeutic strategies for obesity and inflammation management. Clinical evidence exists for phenolic compounds in preventing and treating cardiometabolic disease (Table 3). However, more preclinical and clinical studies are still required to demonstrate the modulation of the gut microbiota through the intake of polyphenols as a therapeutic target or preventive measure for cardiometabolic pathologies.

### 4.5. Current State of Use of Phenolic Compounds in Gut Microbiota and Cardiovascular Health

Promising results have emerged from observational studies on phenolic compounds, yet alternatives are limited to synthesized derivatives awaiting clinical trial outcomes [152]. For instance, synthesized compounds like TensioFytol^®^, which includes olive dry extract rich in hydroxytyrosol and oleuropein, demonstrate potent antioxidative properties. The results of TensioFytol^®^ are currently awaiting publication under the clinical trial number NCT04874961.

The Mediterranean diet (MedDiet), renowned for reducing cardiovascular disease (CVD) risk, emphasizes the consumption of polyphenol-rich foods such as extra virgin olive oil, nuts, and fruits [153,154]. Despite evidence showing that phenolic compounds improve cardiovascular health, their low bioavailability in the small intestine means most reach the colon, undergoing enzymatic transformations by the gut microbiota [85,155]. These microbial phenolic metabolites (MPMs) formed in the colon may exert significant biological effects. A five-year clinical trial indicates that MPMs can influence gut health by modulating the composition and function of the microbiota, which in turn impacts systemic inflammation and cardiovascular health [156]. However, research specifically assessing the cardiovascular benefits of various classes of MPMs is still limited [155]. Additionally, one aspect that must be considered when talking about the Mediterranean diet would be the restriction of caloric intake, which is often overlooked and could be a confounding factor that can affect the effects of phenolic compounds and the results of these studies.

The interaction between polyphenols and the gut microbiota presents a complex but promising area of study, suggesting that optimizing polyphenol intake and enhancing their bioavailability could lead to better cardiovascular outcomes [157]. While clinical trials on synthesized phenolic compound derivatives are awaited, a systematic review found differences in terms of the details provided to the total and individual phenolic compounds intake between five clinical studies selected; therefore, these differences may affect the results in health observed [158]. Even though there is potential for phenolic compounds to not only act as direct antioxidants but also modulate the gut microbiota and subsequent metabolic pathways, there is not enough clinical evidence to determine these significant effects in promoting cardiovascular health [159]. Given the increasing prevalence of cardiovascular diseases, integrating phenolic compounds into dietary interventions remains a key focus. Consequently, future research should aim to elucidate these mechanisms further, paving the way for more effective dietary strategies and therapeutic applications.

## 5. Role of Phenolic Compounds in Gut Microbiota Production of Neurotransmitters and Cardiometabolic Health

Cardiovascular disease represents a major health challenge, and many pathways are being studied to understand and find novel ways to treat it, even in neuro-modulation [160]. Serotonin (5-hydroxytryptamine, 5-HT), a biogenic amine that functions as both a neurotransmitter and a peripheral hormone, plays a role in the development of cardiovascular disease [161]. Phenolic compounds, bioactive compounds found in many plants, have demonstrated significant health benefits, particularly for cardiovascular health. These substances influence neurotransmitter metabolism, enhance the functionality of neurotransmitter receptors, and bolster antioxidative defenses [162,163]. By modulating these pathways, polyphenols show promise as therapeutic agents for preventing and managing cardiovascular diseases, offering a natural approach to improving heart health and reducing the risk of cardiovascular events.

By promoting the growth of bacteria that are beneficial to host health, flavonoids stimulate the production of various metabolites, such as short-chain fatty acids (SCFAs), γ-aminobutyric acid (GABA), and brain-derived neurotrophic factor (BDNF). Some of these metabolites can be biologically transformed into neurotransmitters [164]. For instance, certain Gram-positive gut bacteria, including *Lactobacillus* spp. and *Bifidobacteria* spp., can convert glutamate into GABA, the primary inhibitory neurotransmitter in the central nervous system [165]. In contrast, phenolic compounds encourage beneficial bacteria, such as *Streptococcus* spp., *Escherichia* spp., and *Enterococcus* spp. genera, to produce a variety of metabolites, including SCFAs, and promote the secretion of hormones and neurotransmitters (serotonin through the metabolism of tryptophan and γ-aminobutyric acid) [166,167,168,169].

Serotonin has a broad spectrum of functions in the cardiovascular system, including roles in atherosclerosis, myocardial infarction, heart failure, thrombosis, and arterial hypertension [161]. However, the clinical or experimental data on the cardiovascular effects of serotonin are partly conflicting, and further research is needed to characterize these effects in specific tissues for targeted pharmacological therapies. Given the involvement of serotonin in cardiovascular diseases, the neuroprotective actions of plant polyphenols in regulating neurotransmitter metabolism and their antioxidative properties hold promise for therapeutic applications. By increasing serotonin levels and improving neurotransmitter balance, phenolic compounds could be a viable and natural alternative for managing cardiovascular conditions.

A proposed hypothesis suggests a possible relationship between the alteration of the gut microbiota by serotonin and the metabolic pathway that leads to insulin resistance, potentially contributing to the etiopathogenesis of diabetes mellitus [170]. Research has shown that type 1 diabetes (DM1) is linked to excess pro-inflammatory cytokines near pancreatic B cells. In contrast, type 2 diabetes (DM2) is associated with elevated levels of pro-inflammatory cytokines in the systemic circulation, which could be related to serotonin secretion in the gut [171,172,173]. Gershon et al. have highlighted the critical role of the 5-HT4 receptor complex in maintaining the enteric nervous system, indicating its dual neurodegenerative and neuroprotective effects [174]. Bianco et al. have demonstrated how 5-HT4 agonists can safeguard enteric neurons from oxidative stress [175]. This fact is particularly important, as neurons lost due to inflammation are significantly impacted by oxidative stress [176,177]. Bhattarai et al. have noted that the microbiome can influence serotonin’s pro-inflammatory “sword” function [178]. The gut employs various mechanisms to balance the actions of 5-HT for maintaining transport. Chang and Rao have integrated the role of the gut microbiota in preserving homeostasis and preventing 5-HT imbalances during conditions like diarrhea and inflammatory bowel diseases [179]. A recent article published by Tan et al. concluded that colonic enteroendocrine cells (EECs) regulate the metabolism through their interaction with the gut microbiota of mouse models [180]. Colonic EEC deficiency leads to hyperphagia and obesity.

Furthermore, colonic EEC deficiency results in altered microbiota composition and metabolism, both of which are necessary and sufficient for the development of obesity [180]. The hypothalamic intermuscular layer (HIM) significantly enhances serotonin production in enterochromaffin cells, stimulating primary afferent neurons and activating interneurons. This chain of events triggers the peristaltic reflex, boosting intestinal motility and speeding up gastric emptying, effects that are amplified when the 5-HT receptor is antagonized [181]. The resultant increase in intestinal motility promotes the production of gastrointestinal hormones that regulate glucose metabolism, leading to insulin synthesis, β-cell proliferation, or glucagon release [182]. Moreover, serotonin has been implicated in the lactate signaling cascade, further stimulating β-cell proliferation [183]. These findings underscore the intricate interplay between the gut microbiota, serotonin, and metabolic pathways, highlighting the potential of targeting gut-derived serotonin in therapeutic strategies for DM2. Understanding this relationship better could open new avenues for managing insulin resistance and diabetes through gut microbiota modulation.

## 6. Conclusions

Phenolic compounds have garnered significant attention in recent years due to their potential beneficial effects and future applications in metabolic diseases such as diabetes mellitus, non-alcoholic fatty liver disease, and cardiovascular diseases. These bioactive molecules exhibit antioxidant, anti-inflammatory, antidiabetic, and anticancer properties, leading to numerous studies exploring their role in various metabolic pathways. Evidence suggests that phenolic compounds reduce oxidative stress, decrease glucose absorption, improve fatty acid oxidation, and alleviate inflammation, thus contributing to the mitigation of various metabolic disorders. The underlying mechanisms involve the positive modulation of the gut microbiota, favoring the abundance of beneficial bacteria and suppressing harmful microorganisms. Importantly, this relationship is bidirectional, as the microbiota plays a crucial role in the metabolism of phenolic compounds, significantly influencing their bioavailability, transformation, and potency. However, most of the supporting evidence for phenolic compounds in cardiometabolic diseases comes from preclinical studies. Therefore, more clinical evidence is needed to demonstrate the modulation of the gut microbiota by phenolic compounds. The current evidence represents the beginning of potential hypotheses that can be further developed to elucidate the role of phenolic compounds in the microbiota and their impact on cardiometabolic diseases. Future research should focus on the consumption of phenolic compounds as a treatment or prophylactic measure for these diseases.

## Figures and Tables

**Table 2:** Preclinical evidence of the implications of polyphenol–gut microbiota interactions in cardiometabolic diseases.

Disease	Polyphenol	Metodology	Results	Ref.
NON-ALCOHOLIC FATTY LIVER DISEASE	Resveratrol	A murine model of NAFLD was established using an HFD feeding regimen. Resveratrol was then administered to the HFD-fed mice via forced feeding to assess its effects on various parameters associated with NAFLD.	Resveratrol reduced hepatic steatosis and insulin resistance in non-alcoholic fatty liver disease (NAFLD). Furthermore, it induced significant alterations in gut microbiota diversity and composition; *Firmicutes* and *Actinobacteria* phyla were increased after resveratrol; contrarily, *Bacteroidetes* were decreased	[105]
	CGA	HFD-induced NAFLD mice were treated with or without CGA. Serum transaminases, fasting glucose, lipids, insulin, GLP-1, and LPS were measured. Hepatic histology was evaluated. The TLR4 pathway was analyzed, and inflammatory cytokines were detected. The gut microbiota was determined, and intestinal protein expressions were examined.	CGA reduced hepatic steatosis and inflammation, serum transaminases, blood glucose and lipids, improved insulin sensitivity, and reversed hepatic TLR4, TNF-α, and IL-6 activation. It also modulated the gut microbiota, increasing Bifidobacterium and decreasing *E. coli*. CGA improved intestinal tight junction protein expression and increased portal vein LPS and GLP-1 levels.	[106]
DIABETES MELLITUS	CGA	The effects of CGA on hepatic lipid metabolism were evaluated in diabetic db/db mice, with db/m mice serving as controls. The db/db mice were assigned to the following treatment groups: (1) DB, received 0.1 mL of PBS once daily; (2) CA, 0.25 g of CGA/kg body weight once daily; (3) MET, 0.25 g of metformin/kg body weight once daily.	Compared to DB, CA reduced body weight and improved glucose tolerance and insulin resistance, decreased hepatic lipid content, downregulated inflammatory gene expression such as TNF-α, IL-1β, and IL-6, upregulated IL-10, and promoted antioxidant genes including SOD1, SOD2, and GPX1, similar to the MET group. Additionally, CGA improved cecal bacterial diversity, restoring the abundance of *Bacteroidetes* and the genera *Lactobacillus*, *Blautia*, and *Enterococcus*, compared to the DB group.	[107]
	Resveratrol and quercetin	Wistar rats were randomly allocated into four groups. These groups were fed with an HFS diet, supplemented or not with trans-resveratrol (15 mg/kg body weight/day), quercetin (30 mg/kg body weight/day), or a combination of both polyphenols at these doses.	Concomitant intake of trans-resveratrol and quercetin acted synergistically to reduce weight gain. Furthermore, administering both polyphenols individually and in combination improved basal insulin levels and the HOMA-IR index. It was also observed that quercetin supplementation positively affected gut microbiota composition, particularly by attenuating the *Firmicutes*/*Bacteroidetes* ratio and inhibiting the growth of *Erysipelotrichaceae*, *Bacillus*, and *Eubacterium cylindroides*.	[108]
CARDIOVASCULAR DISEASE	Resveratrol	The role of the intestinal microbiota in the protective effects of resveratrol against AS in mice was determined.	Resveratrol mitigates TMAO-induced atherosclerosis by reducing TMAO levels and enhancing hepatic bile acid neosynthesis via gut microbiota modulation. Moreover, bile acid neosynthesis and the FXR-FGF15 enterohepatic axis were affected.	[109]

Abbreviations: NAFLD: non-alcoholic fatty liver disease; HFD: high-fat diet; TAG: triacyl-glycerols; LDL-C: low-density lipoprotein cholesterol; AGCC: short-chain fatty acids; GPR43: G protein-coupled receptor 43; NF-κB: nuclear factor kappa B; TNF-α: tumor necrosis factor alpha; TLR4: toll-like receptor 4; IL-6: interleukin-6; LPS: lipopolysaccharides; GLP-1: glucagon-like peptide-1; CGA: chlorogenic acid; DB: double-blind group, animals were orally gavaged with 0.1 mL PBS once a day; MET: metformin group, animals were gavaged with 0.25 g metformin/kg body weight once a day; CA: chlorogenic acid group, animals were gavaged with 0.25 g CGA/kg body weight once a day; IL-1β: interleukin 1 beta; IL-10: interleukin 10; SOD1: superoxide dismutase type 1; SOD2: superoxide dismutase type 2; GPX1: glutathione peroxidase 1; HFS: high-fat–high-sugar diet; HOMA-IR: homeostatic model assessment for insulin resistance; AS: atherosclerosis; TMAO: trimethylamine-N-oxide; FXR: nuclear receptor farnesoid X receptor; FGF15: fibroblast growth factor 15.

**Table 3:** Clinical evidence for the implications of polyphenol and gut microbiota interaction in cardiometabolic diseases.

Disease	Polyphenol	Methodology	Results	Ref.
NON-ALCOHOLIC FATTY LIVER DISEASE	Resveratrol	A randomized controlled trial was conducted with 90 patients (men and women) aged 20 to 60 years with NAFLD and a BMI of 25 to 35 kg/m^2^. They were randomly assigned to three groups: low-calorie diet (n = 30), 600 mg daily resveratrol (n = 30), or placebo (n = 30) for 12 weeks.	A significant reduction in weight (1.1%) and BMI was found in the resveratrol group compared to the placebo (*p* < 0.05). ALT, AST, and lipid profiles did not change in the resveratrol group (all *p* > 0.05). There were no significant changes in hepatic steatosis, glycemic parameters, HDL cholesterol levels, and sirtuin-1 in any group (*p* > 0.05).	[113]
	Curcumin, resveratrol, naringenin, anthocyanin, hesperidin, catechin, silymarin, and genistein.	In this meta-analysis, an exhaustive literature search was conducted in Chinese and English up to 30 April 2022, using databases such as PubMed, Cochrane, Medline, CNKI, and others to examine the treatment of NAFLD with dietary polyphenols.	Curcumin may decrease BMI, AST, ALT, TG, TC, and HOMA-IR compared to placebo. Naringenin significantly decreased LDL-C and NAFLD, TG, and TC and increased HDL-C but had no significant effect on AST and ALT. Hesperidin may decrease BMI, AST, ALT, TG, TC, and HOMA-IR. Catechin may decrease BMI, HOMA-IR, and TG levels. Silymarin effectively improved ALT and AST and reduced hepatic fat accumulation and liver stiffness in patients with NAFLD.	[114]
	Resveratrol	In this randomized, double-masked, placebo-controlled trial, 50 patients with NAFLD received a 500 mg resveratrol or placebo capsule supplement for 12 weeks.	Resveratrol supplementation significantly reduced ALT levels and hepatic steatosis compared to the placebo (*p* < 0.05). No significant changes were observed in either group’s blood pressure, insulin resistance markers, or TG levels (*p* > 0.05).	[115]
DIABETES MELLITUS	Resveratrol	A randomized, double-masked, placebo-controlled, parallel-group trial was conducted. One hundred and ten diabetic patients participated and were randomly assigned to two groups: one received 200 mg of resveratrol daily (n = 55), while the other received a placebo (n = 55), for a period of 24 weeks, after providing informed consent.	Resveratrol significantly reduced plasma glucose, insulin, HOMA-IR, malondialdehyde, high-sensitivity C-reactive protein, TNF-α, and IL-6. Additionally, a greater than two-fold downregulation was observed in the expression of miR-34a, miR-375, miR-21, and miR-192 as well as an upregulation in the expression of miR-126 and miR-132.	[111]
	Tea	This cohort study utilized data from the China Health and Nutrition Survey, which involved 5199 participants initially recruited in 1997 and followed-up until 2009.	High tea consumption (e.g., ≥4 cups/day) was associated with a reduced risk of TDM.	[116]
	Resveratrol	A randomized, controlled, single-masked, parallel-group trial. It included 472 elderly patients with type 2 diabetes mellitus assigned to two groups: one received resveratrol (n = 242) and the other a placebo (n = 230) for six months.	Resveratrol reduced blood glucose levels and improved the lipid profile and renal function compared to the placebo. It reduced the production of glucose-6-phosphatase, HbA1c, IL-6, TNF-α, and IL-1β in elderly patients with diabetes.	[112]
	Quercetin	In a parallel, randomized, placebo-controlled, double-blind study, eighty-eight post-MI patients were recruited from Rasool-e-Akram and Afshar hospitals in Iran. Participants with a body mass index ≤ 35 kg/m^2^ and aged between 30 and 65 years were randomly assigned to receive a daily dose of 500 mg of quercetin (n = 44) or placebo (n = 44) for 8 weeks.	In post-MI patients, the daily administration of 500 mg of quercetin for 8 weeks did not affect biomarkers of endothelial dysfunction.	[117]
	Quercetin	In this meta-analysis, searches were conducted in the Medline, EMBASE, and Web of Science databases to identify randomized controlled trials evaluating the impact of quercetin on BP up to May 2022.	Quercetin significantly decreased SBP in the mixed population and the normotensive subgroup and DBP in the pre-hypertensive subgroup.	[118]
CARDIOVASCULAR DISEASE	Polyphenols of red wine	A controlled, randomized, crossover intervention study was conducted with ten healthy male volunteers recruited. After a washout, each subject received red wine, the same amount of de-alcoholized red wine, or gin for 20 consecutive days.	The consumption of red wine polyphenols produced significant reductions in SBP and DBP as well as in TG, total cholesterol, HDL cholesterol, and C-reactive protein (*p* < 0.05). Additionally, it increased the presence of bacterial groups such as *Enterococcus*, *Prevotella*, *Bacteroides*, *Bifidobacterium*, *Bacteroides uniformis*, *Eggerthella lenta* and *Blautia coccoides-Eubacterium rectale* (*p* < 0.05) compared to baseline values.	[95]

Abbreviations: NAFLD: non-alcoholic fatty liver disease, HFD: high-fat diet, TAG: triacylglycerides, HDL: high-density lipoproteins, LDL-C: low-density lipoproteins, NF-κB: nuclear factor kappa B, TNF-α: tumor necrosis factor alpha, IL-6: interleukin-6, IL-1β: interleukin-1 beta, IL-10: interleukin-10, HOMA-IR: homeostatic model assessment for insulin resistance, AS: atherosclerosis, HbA1c: glycated hemoglobin, SBP: systolic blood pressure, DBP: diastolic blood pressure, MI: myocardial infarction, T2DM: type 2 diabetes mellitus, CNKI: China National Knowledge Infrastructure, miRNA: microRNA, BMI: body mass index, AST: aspartate aminotransferase, ALT: alanine aminotransferase, TC: total cholesterol.
